# Cost of illness for outpatients attending public and private hospitals in Bangladesh

**DOI:** 10.1186/s12939-016-0458-x

**Published:** 2016-10-10

**Authors:** Md Sadik Pavel, Sayan Chakrabarty, Jeff Gow

**Affiliations:** 1 Department of Economics, Shahjalal University of Science & Technology, Sylhet, 3114 Bangladesh; 2 Institute for Resilient Regions (IRR), University of Southern Queensland, Springfield, 4300 QLD Australia; 3 School of Commerce, University of Southern Queensland, Toowoomba, 4350 QLD Australia; 4 School of Accounting, Economics and Finance, University of KwaZulu-Natal, Durban, 4000 South Africa

**Keywords:** Total cost of outpatients, Direct cost, Indirect cost, Health care, Public vs private, Bangladesh

## Abstract

**Background:**

A central aim of Universal Health Coverage (UHC) is protection for all against the cost of illness. In a low income country like Bangladesh the cost burden of health care in tertiary facilities is likely to be significant for most citizens. This cost of an episode of illness is a relatively unexplored policy issue in Bangladesh. The objective of this study was to estimate an outpatient’s total cost of illness as result of treatment in private and public hospitals in Sylhet, Bangladesh.

**Methods:**

The study used face to face interviews at three hospitals (one public and two private) to elicit cost data from presenting outpatients. Other socio-economic and demographic data was also collected. A sample of 252 outpatients were randomly selected and interviewed. The total cost of outpatients comprises direct medical costs, non-medical costs and the indirect costs of patients and caregivers. Indirect costs comprise travel and waiting times and income losses associated with treatment.

**Results:**

The costs of illness are significant for many of Bangladesh citizens. The direct costs are relatively minor compared to the large indirect cost burden that illness places on households. These indirect costs are mainly the result of time off work and foregone wages. Private hospital patients have higher average direct costs than public hospital patients. However, average indirect costs are higher for public hospital patients than private hospital patients by a factor of almost two. Total costs of outpatients are higher in public hospitals compared to private hospitals regardless of patient’s income, gender, age or illness.

**Conclusion:**

Overall, public hospital patients, who tend to be the poorest, bear a larger economic burden of illness and treatment than relatively wealthier private hospital patients. The large economic impacts of illness need a public policy response which at a minimum should include a national health insurance scheme as a matter of urgency.

**Electronic supplementary material:**

The online version of this article (doi:10.1186/s12939-016-0458-x) contains supplementary material, which is available to authorized users.

## Background

The health of the people of Bangladesh has improved in recent years. This is evidenced by reductions in infant and child mortality rates, increased vaccination rates, increased availability of birth control, reduction in cholera prevalence and improved arsenic prevention [1]. Over the past 20 years health care availability has increased as has the cost of treatment. Individuals’ expenditure on health care has increased as a result. Cost barriers however still prevent the poorest of the poor from accessing health care [2]. According to the Bangladesh Bureau of Statistics [3] in 2010, 15 % of sick people were not treated due to their inability to pay for the (relatively) high cost of health care. Detailed cost of illness studies make a significant contribution to understanding the differential cost burden of illness [4, 5].

Bangladesh has a mixed health care system with both public and private providers of primary health care and outpatient services through tertiary hospitals. Bangladesh is a low income country and in the face of inadequate public health care expenditure, health care providers have adopted the pre-payment mechanism where individuals must pay for treatment before receiving it. This is a barrier to health care because of the relatively high costs involved [6, 7]. In low income countries households spend up to 40 % of their incomes on health care, whereas that figure is less than 20 % for middle and high income countries [8–11]. Thus the large financial burden of health care is borne by the poorest of society [9–12].

A recent International Center for Diarrhoeal Disease Research, Bangladesh (ICDDR’B) study revealed that around 6.4 million or 4 % of people in Bangladesh get poorer every year due to excessive health costs [13]. It found that the poorest 20 % of the population spent 16.5 % of their household income on direct health care costs, while the richest 20 % spent just 9.2 %. Out of pocket health expenditure by households totaled 64 % of direct costs with the rest coming from government and other sources [13]. This is an unreasonable burden for many households in a nation with an average per capita income of just on $US1000 per year [3].

This current study aims to inform policy makers about the costs, both direct and indirect, of outpatient treatment in public and private hospitals in one city in Bangladesh. Given low incomes the financial burden of health care is beyond the means of many people. This results in significant numbers of people receiving inadequate treatment for illnesses or worse receiving no health care at all, due to the insurmountable financial burden of its cost. The results of this study will inform those organizations trying to achieve Universal Health Coverage (UHC) in Bangladesh. The WHO (2010) defines UHC as access to good quality health care services where people do not suffer unreasonable financial hardship to pay for them [7, 14–17]. Research on the cost of illness is required to inform the development of appropriate social policies to improve access to essential health services and break the vicious cycle between illness and poverty [10]. Therefore, an analysis of total (direct and indirect) costs of outpatients in both the public and private hospital sectors is extremely important. It will assist Bangladeshi policy makers to develop alternative methods to protect individuals and households from the extreme and catastrophic financial burden of illness and health care treatment and assist to increase access to health care services.

The purpose of the study is to calculate the total cost of illness for outpatients due to different types of illnesses in public and private hospitals in Sylhet, Bangladesh. This study defines the direct costs of treatment (such as fees, medications) and indirect costs of illness (such as travel time and loss of income) of outpatients for different types of illness using established and validated cost methodologies [4, 14].

## Methods

### Study area

**Fig. 1:**
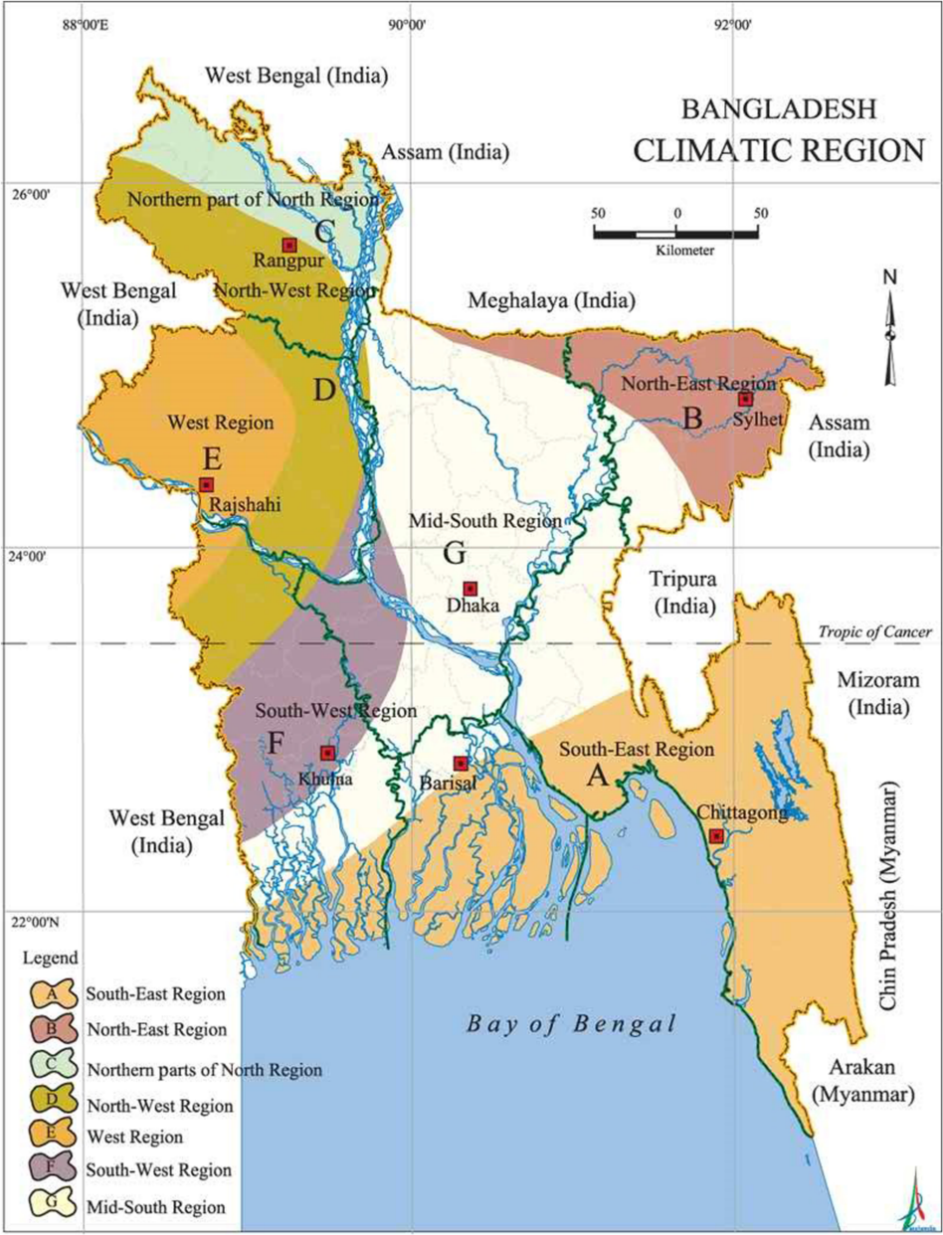
Region specified map of Bangladesh. Source: Banglapedia - National encyclopedia of Bangladesh 2011 (http://en.banglapedia.org/index.php?title=Climate Accessed on 28^th^ May 2016)

**Fig. 2:**
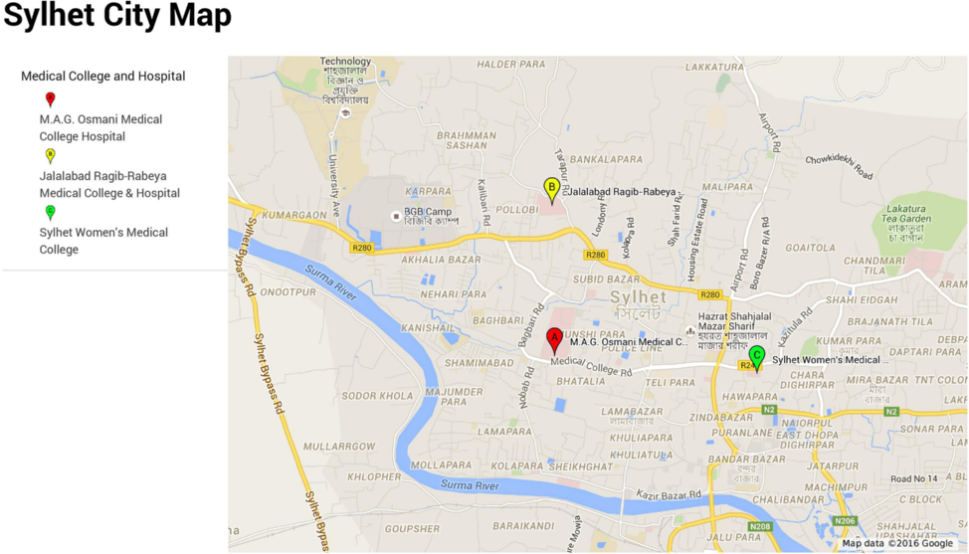
Sylhet City Map. Source: Google Maps 2016

The divisional city of Sylhet (a major city in north-eastern Bangladesh) which is situated in north-eastern of Bangladesh was purposefully selected (Fig. 1). As a divisional city, people from surrounding areas also received health care in Sylhet. The city was chosen as it has one public and three private medical training colleges and public hospitals and many private primary health care clinics [18]. Data were collected in 2011 via face to face interviews with a total of 252 outpatients from one public medical college (MAG Osmani Medical College Hospital) and two private medical college hospitals (Jalalabad Ragib Rabeya Medical College and Hospital and the Women’s Medical College and Hospital) (Fig. 2).

### Participants, procedures and ethical clearance

Patients were randomly selected and interviewed immediately after their consultation. A serial number was assigned to each patient before their consultation and patients were randomly chosen. The random sample of patients avoided sample selection bias and also any potential identification problem. Enumerators waited outside the doctor’s office for the randomly assigned patient to exit. Any patient who came for treatment was eligible to take part in the study.

A structured questionnaire was administered to patients. This was designed to collect data including components of direct medical and non-medical costs, indirect costs, illness details and details of their socio-economic status. The questionnaire is shown in Additional file 1. These data were supplemented with data from hospital staff on some direct costs and informal payments.

Enumerators provided some initial basic information to patients about the study to get their agreement and cooperation. No inducement, financial or otherwise, was offered. Verbal informed consent was obtained before proceeding with the interview. When the patient was a child (below the age of 14) the accompanying adult person answered the questionnaire. Ten enumerators (university students) were trained to administer the questionnaire.

The ethics committee of the Medical Faculty, Shahjalal University of Science & Technology, approved the study, reference number 570-2007/11.

### Measuring the cost of Illness

The total cost of an outpatient’s illness includes direct, indirect and intangible costs [19]. Direct costs are the range of financial costs of health provider services, medicines and other related observable costs. Indirect costs are the monetary value of productive time losses to the patient and other family members as a result of the illness [10]. Intangible costs relate to suffering and grief from illness and are not generally measurable due to their subjective nature [19, 20]. In this study, the intangible costs of illness were not considered.

### Direct costs

Direct costs includes medical and non-medical costs; medical costs include diagnosis, registration fees, medications, diagnostics, continuing care, hospitalization, rehabilitation; and non-medical costs are the costs of transport to the hospital and any informal payments [21, 22]. Informal payments are defined as a money transfer from patient to hospital staff with the expectation of quick or better treatment [23]. The informal payments and medicine cost information were collected from patients during the interview though those were not included in the formal questionnaire. When the patient spoke about informal payments (bribes) to hospital staff, the enumerators asked about the amount and wrote it beside the related section. A similar method was employed for the medicine costs. These payments were cross checked with staff and the patient values were utilized in the analysis.

### Calculating the indirect costs of illness

Indirect costs of illness are those related to income or productivity loss. This is the monetary value of a patient or family caregiver’s income lost due to illness related absences from work (both paid and unpaid) [21, 24]. Household’s loss of work time or productivity are significantly affected by illness type [25]. These losses can be valued from either the societal, individual/household or employer perspectives [26]. An individual/household perspective is adopted in this study.

There are different approaches to measuring total productivity losses due to illness and most studies are based on human capital theory. The human capital approach or friction cost method estimates the value of potential production losses (or income loss as a proxy) as a consequence of illness [27–29]. Self-reported wage rates have been used. Indirect cost was calculated for both paid and unpaid work (care giving, household activities). The income loss from foregone non-market activities (unpaid work) was measured using occupation specific wages [29].

### Data analysis

Data were analyzed using SPSS 20. All entries were double checked. Independent-sample t tests and one-way ANOVA tests were used to analyze if the outlined differences in direct and indirect costs in public and private hospitals were statistically significant. Costs were presented as an average with a standard deviation in the local currency, Bangladeshi Taka (BDT). US dollar (US$) values were also reported using the exchange rate of US$1 = 75 BDT obtained from the Central Bank of Bangladesh during the mid-point of the data collection year (2011).

## Results

The objective of this study was to estimate patient’s total cost (direct and indirect) of treatment and compare individual cost components between private and public hospitals in Bangladesh. This section outlines the cost burden of disease by gender, age group, income quintile, disease type, and treatment modality in both public and private hospital.

### Descriptive statistics

A total of 252 respondents participated in this study with 139 attending the public hospital and 113 attending the two private hospitals. The results in Table 1 present descriptive statistics on respondent’s characteristics: the mean age of respondents both in public and private hospital were almost similar. The average monthly income of public hospital respondents was half that of private hospital respondents. This indicates a common bias of higher income people obtaining health care from private hospitals in preference to public hospitals. Villagers from rural areas, who tend to be poorer than city dwellers go to public hospitals more than the city dwellers and overall 72 % of public hospital respondents came from villages.

**Table 1:** Respondents Characteristics

	Public hospital (*N* = 139)		Private hospital (*N* = 113)	
Mean Age (S.D)	33.55 (20.10)		33.76 (20.05)	
Mean monthly income (S.D)	BDT	10969 (9822)	BDT	20252 (15108)
	US$	146.27 (130.96)	US$	270.03 (201.44)
Sex (Female) %	61 (43.9 %)		50 (44.2 %)	
Living Location (Village) %	100 (71.9 %)		50 (44.2 %)	

Note: 1US $ = 75 BDT as June 2011

Table 2 demonstrates that the average direct cost of treatment for illness was marginally more for public than for private hospital patients. Direct costs in both were less than 4 % of overall total costs. The most significant direct cost issue for public patients were average transport costs and average informal payments which were much higher than for private patients. Average indirect cost or patient’s income loss were the most significant costs which in public hospital was 97 % of total costs and 95 % in private hospital patients. Results from Table 2 indicate that public hospital patients on average paid more for their health care compared to private hospital patients despite being poorer.

**Table 2:** Average cost of treatment by hospital type and treatment modality, BDT (US$)

Cost	Parameters	Public hospital (*N* = 139)			Private hospital (*N* = 113)		
		Average cost BDT ($US)	Standard deviation BDT ($US)	Proportion of total cost (%)	Average cost BDT ($US)	Standard deviation BDT ($US)	Proportion of total cost (%)
Direct Medical	Diagnostic	123 (1.65)	101 (1.35)	1.24	151 (2.02)	137 (1.83)	2.70
	Medicine	29 (0.39)	21 (0.29)	0.29	28 (0.38)	5 (0.07)	0.50
	Registration	21 (0.29)	19 (0.25)	0.22	37 (0.50)	34 (0.46)	0.67
Direct Non-medical	Transport	73 (0.98)	57 (0.77)	0.74	43 (0.59)	32 (0.43)	0.78
	Informal payment	31 (0.41)	31 (0.42)	0.31	8 (0.12)	14 (0.19)	0.15
Total Direct Cost		279 (3.72)	146 (1.95)	2.81	269 (3.59)	163 (2.18)	4.81
Indirect Cost	Patient’s income loss	9643 (128.59)	9296 (123.95)	97.19	5338 (71.18)	6590 (87.87)	95.19
Total Cost of Treatment		9923 (132.31)	9335 (124.47)	100	5607 (74.77)	6562 (87.49)	100

Note: 1US $ = 75 BDT as at June 2011

The analysis in Table 3 shows that the average total costs for public hospital patients were higher than private patients across all income quintiles. Costs for the lowest income public patients were the second highest of any income quintile, either public or private. That is, those with the least capacity to pay are paying the highest costs of illness and treatment. Average indirect cost analysis in Table 3 shows that patients treated in public hospital paid more for their health care across all income quintiles.

**Table 3:** Average cost of treatment by income quintile, BDT (US$)

Income quintile BDT (US$)	Public hospital (*N* = 139)				Private hospital (*N* = 113)			
	*N*	Average direct cost	Average indirect cost	Average total cost	*N*	Average direct cost	Average indirect cost	Average total cost
<6212 (<82.82)	52	282 (3.76)	10683 (142.45)	10966 (146.21)	13	270 (3.61)	3763 (50.18)	4033 (53.78)
6212–12424 (82.82–165.65)	53	280 (3.74)	8658 (115.44)	8938 (119.18)	36	245 (3.27)	5134 (68.48)	5379 (71.73)
12425–18637 (165.66–248.49)	14	194 (2.59)	7980 (106.41)	8174 (108.99)	14	244 (3.26)	4898 (65.32)	5143 (68.57)
18638–24849 (248.50–331.32)	11	326 (4.35)	12059 (160.79)	12385 (165.14)	13	236 (3.16)	8564 (114.20)	8801 (117.36)
≥24850 (≥331.33)	9	327 (4.37)	9076 (121.03)	9404 (125.39)	37	313 (4.19)	5122 (68.301)	5436 (72.49)

Note: 1US $ = 75 BDT as at June 2011

The total costs of treatment by age quintiles (Table 4) show a similar pattern with public patients at all age levels paying more than private hospital patients. Costs rise in line with age in both cohorts. Average direct cost was low compared to the average indirect cost for each age quintile in both public and private hospitals. The average direct cost analysis in Table 4 shows that patients treated in public hospital spend more money in each age quintile except 60 plus age. The average indirect cost analysis suggests that patients treated in public hospital faced more income or productivity loss in each age quintile than that of private hospital patients. From the above discussion the total costs of illness were much higher up to the third age quintile (36 to 60) for public hospital’s patients but were higher for the last age quintile (60 plus) for private hospital’s patients.

**Table 4:** Average cost of treatment by age group, BDT (US$)

Age group (Years)	Public hospital (*N* = 139)				Private hospital (*N* = 113)			
	*N*	Average direct cost	Average indirect cost	Average total cost	*N*	Average direct cost	Average indirect cost	Average total cost
Up to 14	25	269 (3.60)	3993 (53.24)	4262 (56.84)	17	241 (3.22)	3768 (50.25)	4009 (53.46)
15 to 35	58	285 (3.81)	9699 (129.33)	9984 (133.13)	54	275 (3.67)	5137 (68.50)	5412 (72.17)
36 to 60	37	263 (3.51)	13367 (178.24)	13631 (181.75)	28	250 (3.34)	5444 (72.59)	5694 (75.93)
60 plus	19	303 (4.05)	9658 (128.77)	9961 (132.82)	14	320 (4.28)	7806 (104.08)	8126 (108.36)

Note: 1US $ = 75 BDT as at June 2011

The losses associated with children illness and adult care of them were significant as shown elsewhere [20].

In the public hospital the average total costs for males and females were higher than for public hospital patients. The analysis in Table 5 shows that average total costs of treatment for illness was higher in public hospital (BDT 9923 or $132.31) than that of private hospital (BDT 5607 or $74.77), regardless of patient’s gender but average direct cost was higher for females in both public and private hospitals. In addition, average indirect cost was higher for both males and females patients in public hospital.

**Table 5:** Cost of treatment by gender, BDT (US$)

Gender	Public hospital (*N* = 139)				Private hospital (*N* = 113)			
	*N*	Average direct cost	Average indirect cost	Average total cost	*N*	Average direct cost	Average indirect cost	Average total cost
Male	78	263 (3.51)	10027 (133.70)	10290 (137.21)	63	242 (3.24)	6074 (81.00)	6317 (84.23)
Female	61	299 (3.99)	9153 (122.05)	9452 (126.04)	50	303 (4.04)	4410 (58.81)	4713 (62.85)
Total	139	279 (3.72)	9643 (128.59)	9923 (132.31)	113	269 (3.59)	5338 (71.18)	5607 (74.77)

Note: 1US $ = 75 BDT as at June 2011

Amongst children (under 14 years of age), analysis of total cost of treatment for illness is presented in Table 6. In public hospital the average total costs for male children were higher than in private hospital. However, this pattern was reversed for girl children treatment. However, for female children, total costs of illness in private hospital were higher than public hospital. These differentials may reflect the alternative attitudes towards girls in poorer compared to richer households and their potential future role as care givers to their parents.

**Table 6:** Gender differential in cost of treatment among children

Gender	Public hospital, BDT (US$)				Private hospital, BDT (US$)			
	*N*	Average direct cost	Average indirect cost	Average total cost	*N*	Average direct cost	Average indirect cost	Average total cost
Male	16	287 (3.83)	4995 (66.61)	5282 (70.44)	12	218 (2.92)	3422 (45.63)	3641 (48.55)
Female	9	238 (3.18)	2211 (29.48)	2449 (32.66)	5	294 (3.92)	4600 (61.33)	4894 (65.26)
Total	25	269 (3.60)	3993 (53.24)	4262 (56.84)	17	241 (3.22)	3768 (50.25)	4009 (53.46)

Note: 1US $ = 75 BDT as at June 2011

Table 7 summarizes the total costs of illness by different disease types and specialized hospital departments. The average total costs do not have a consistent pattern across public and private hospitals. In fact much heterogeneity is evidenced especially direct costs. As such the results should be accepted but with caution. The analysis in Table 7 indicates that the total costs of treatment by illness varied across all hospital departments both in public and private hospitals. The direct costs of treatment for illness were higher in all hospital departments in public hospital than private hospital except surgery, gynecology, and orthopedics. Indirect costs of treatment for illness was also higher for public hospital patients except medicine, chest medicine, orthopedics, and rheumatology departments compared to private hospital patients.

**Table 7:** Cost of treatment by department

Department of hospital	Public hospital, BDT (US$)				Private hospital, BDT (US$)			
	*N*	Average direct cost	Average indirect cost	Average total cost	*N*	Average direct cost	Average indirect cost	Average total cost
Surgery	11	279 (3.73)	11999 (159.99)	12278 (163.72)	2	738 (9.85)	6250 (83.33)	6988 (93.18)
Skin	8	261 (3.48)	7630 (101.74)	7891 (105.22)	11	164 (2.19)	3497 (46.63)	3661 (48.82)
Medicine	25	292 (3.90)	4806 (64.08)	5098 (67.98)	42	241 (3.22)	5510 (73.48)	5752 (76.69)
Ear, Nose and Throat	9	306 (4.09)	12921 (172.28)	13228 (176.38)	2	285 (3.81)	250 (3.33)	535 (7.14)
Neurology	8	259 (3.46)	9455 (126.07)	9715 (129.54)	5	244 (3.26)	1460 (19.47)	1704 (22.73)
Gynecology	16	345 (4.61)	11630 (155.08)	11976 (159.69)	11	555 (7.41)	2022 (26.97)	2578 (34.38)
Cardiology	20	321 (4.29)	11963 (159.51)	12284 (163.80)	6	265 (3.54)	8431 (112.41)	8696 (115.96)
Chest Medicine	4	303 (4.04)	10838 (144.51)	11141 (148.55)	3	200 (2.68)	11550 (154.01)	11751 (156.69)
Orthopedics	19	178 (2.38)	13444 (179.26)	13622 (181.64)	1	211 (2.82)	24000 (320.00)	24211 (322.82)
Rheumatology	2	267 (3.57)	3150 (42.00)	3417 (45.57)	5	255 (3.40)	6293 (83.91)	6548 (87.31)
Others (Non-specific)	17	258 (3.45)	6171 (82.29)	6430 (85.74)	25	218 (2.91)	600 (80.02)	6219 (82.93)

Note: 1US $ = 75 BDT as at June 2011

The higher indirect costs in public hospital patients is primarily explained by high travel and long waiting times, especially compared to private hospital patients. Public hospital patients spend on average almost double the time accessing treatment which includes travel time and waiting time at the hospital to see a doctor. Table 8 indicates that public hospital patients spend approximately double the time compared to private hospital patients. Most public hospital patients (71 %) were coming from rural areas and their travel time and cost is higher than that of patients who visited private hospitals who mainly resided in the city. In public hospital the numbers of doctors were insufficient and there were always long queues for treatment observed. Some of the public hospital patients tried to jump the queue by offering bribes to staff in an attempt to get to see the doctor more quickly. In public hospital, 114 out of 139 patients (82 %) paid money as informal payments to see the doctor earlier. On the contrary, only 44 out of 113 patients (38 %) paid money as informal payments to private hospitals.

**Table 8:** Travel and waiting time for treatment

Hospital type	*N*	Average time spent (minutes)		
		Travel Time	Waiting Time	Total Time
Public Hospital	139	75.59	72.71	148.30
Private Hospital	113	44.14	38.11	82.25

Some patients in both the public and private hospital also expressed dissatisfaction about treatment and wanted to change their current hospital to access better treatment. The prevalence of this dissatisfaction was higher in the public hospital. In the public hospital, 22 % of patients were interested to change, compared to 8 % among the private patients (Table 9).

**Table 9:** Dissatisfaction with treatment received

	Treated in public hospital and moved to another hospital to receive better treatment	Treated in private hospital and moved to another hospital to receive better treatment
Number of dissatisfied patients	31 out of 139 (22.3 %)	9 out of 113 (8 %)

### Statistical analysis

Independent-sample t tests and one-way ANOVA tests were used to analyze if the outlined differences in direct and indirect costs in public and private hospitals were statistically significant.

Table 10 shows the independent-samples t test results of the group summary statistics of the total direct costs and total indirect costs. For public hospital patients, total direct medical costs and total indirect costs were higher than for private hospital patients. This result is antithetical to an equitable outcome for health care given the income and wealth differentials.

**Table 10:** Independent-sample t test summary statistics

	Nature of the health care	*N*	Mean	Std. deviation	Std. error mean
Total Direct Medical Cost in USD	Public	139	3.722	1.950	0.165
	Private	113	3.593	2.180	0.205
Total Indirect Cost in USD	Public	139	128.585	123.955	10.513
	Private	113	71.176	87.868	8.265

In Table 11 the Levene’s Test for Equality of Variances show that for total direct cost the outcomes are not statistically significant. Further it can be concluded that the means of total direct costs for public and private hospital patients were not significantly different. The mean difference was 0.129, and the p-value is 0.621 which indicates the absolute difference between the two means is about 62 %.

**Table 11:** Independent-sample t test analysis

		Levene’s test for equality of variances		t-test for equality of means						
		F	Sig.	t	df	Sig. (2-tailed)	Mean difference	Std. Error difference	95 % Confidence interval of the difference	
									Lower	Upper
Total Direct Medical Cost in USD	Equal variances assumed	0.020	0.889	0.496	250	0.621	0.129	0.260	−0.383	0.642
	Equal variances not assumed			0.490	227.084	0.625	0.129	0.263	−0.390	0.648
Total Indirect Cost in USD	Equal variances assumed	20.687	0.000	4.148	250	0.000	57.408	13.840	30.149	84.668
	Equal variances not assumed			4.293	245.672	0.000	57.408	13.374	31.066	83.751

The Levene’s Test for Equality of Variances for the total indirect costs indicate statistical significance. This result suggests that variances for the two groups, public and private, were different. The mean difference was 31.06 which suggests that the difference in means is statistically significantly different from zero.

Table 12 shows the results of the one way ANOVA to test the homogeneity of variances for the total direct and total indirect costs. The test assumes that the two variances are the same, that is, H_0_: σ^2^_public_ = σ^2^_private_. For total direct cost it failed to reject H_0_ implying that there was little evidence that the variances were not equal and the homogeneity of variance assumption may be reasonably satisfied. On the contrary, for total indirect cost H_0_ is rejected implying that there was evidence that the variances were equal and the homogeneity of variance assumption may not be reasonably satisfied.

**Table 12:** One way ANOVA test - test of homogeneity of variances

	Levene statistic	df1	df2	Sig.
Total Direct Medical Cost in USD	0.020	1	250	0.889
Total Indirect Cost in USD	20.687	1	250	0.000

Table 13 shows the output of the one way ANOVA analysis indicating whether there were significant differences between group means. The results on total direct medical cost shows that there was no statistically significant difference between public and private hospital patient groups. On the contrary, the one way ANOVA on total indirect medical cost shows there was a statistically significant difference between public and private hospital patient groups.

**Table 13:** One Way ANOVA Test Analysis

		Sum of squares	df	Mean square	F	Sig.
Total Direct Medical Cost in USD	Between Groups	1.039	1	1.039	0.246	0.621
	Within Groups	1057.296	250	4.229		
	Total	1058.335	251			
Total Indirect Cost in USD	Between Groups	205422.193	1	205422.193	17.204	0.000
	Within Groups	2985107.360	250	11940.429		
	Total	3190529.553	251			

Table 14 shows the results of the Robust Test of Equality of Means, which has been conducted using the Welch and Brown-Forsythe method. The result of the total direct medical costs show that there was no statistically significant difference between public and private hospital patient groups. On the contrary, the Welch and Brown-Forsythe test on total indirect medical costs show that there was a statistically significant difference between public and private hospital patient groups.

**Table 14:** One Way ANOVA Test - Robust Test of Equality of Means

		Statistic^a^	df1	df2	Sig.
Total Direct Medical Cost in USD	Welch	0.240	1	227.084	0.625
	Brown-Forsythe	0.240	1	227.084	0.625
Total Indirect Cost in USD	Welch	18.426	1	245.672	0.000
	Brown-Forsythe	18.426	1	245.672	0.000

^a^Asymptotically F distributed

## Discussion

The purpose of this study was to examine the direct and indirect costs of outpatient treatment for different types of illnesses in public and private hospitals in Sylhet, Bangladesh. The direct costs of treatment make up only a small part of the total costs of treatment. However, these direct (monetary) costs are a large burden in the context of extremely low incomes particular for public hospital patients. The majority of the costs however are indirect which are primarily income losses of patients and their caregivers due to illness. The indirect costs are over 95 % for both public and private outpatients of total costs of illness.

Among the individual features: age, gender and disease differences have an effect on the direct, indirect and total costs of illness, whilst outpatients age 60 and over experience the highest direct cost of illness. The average direct cost for female outpatients is higher than male outpatients both in public and private hospitals. The loss of income to parents due to a children illness was significant. Amongst child outpatients female children’s average direct cost is also higher than that of male children in private hospital. Old age patients and females are more vulnerable and negatively affected by fees and associated direct spending for treatment. The divergent social roles assigned to women, men and older people affects accessibility and control over resources and decision-making needed to protect health. This results in inequitable patterns of health services especially when the cost of treatment is higher for women (cost of gynecology is higher any other department) and old age people. Health service delivery should strive for equity, therefore, age and gender sensitive service delivery should be effectively addressed by innovative health policies.

**Fig. 3:**
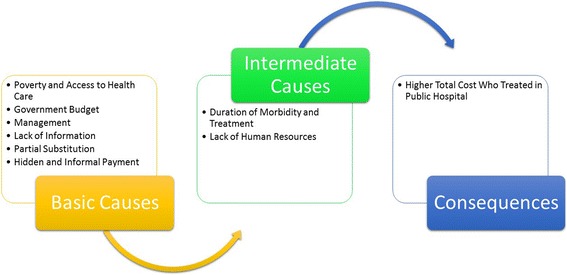
Causes and consequences of public hospital outpatients higher cost

Overall public hospital outpatients experience higher total costs than those treated in private hospital. This is significant and the causes and consequences are shown in Fig. 3. Poverty is the main problem of public hospital outpatients. The relatively high cost of health care services reduces its demand, but not the need for the health care. Usually the poorest outpatients waited the longest to consult a doctor. This is problematic when their conditions have already deteriorated as a result of delaying treatment and the associated financial cost. Medications that are provided in public hospitals are meant to be “free” but are often unavailable. Moreover, poor outpatients regularly substitute doctor care with the local pharmacy owners’ opinion. This can be dangerous because those sellers rarely have any formal education in medicine or pharmaceuticals. Further, the pharmaceutical supply chain in developing countries like Bangladesh are fraught with various problems and put treatments at risk [30]. As a result of these issues, the morbidity of the poor frequently becomes complicated and increases the duration of treatment. This study recommends that more attention be paid to the costs of medication. It is apparent that the present technology infrastructure of Bangladesh’s pharmaceutical companies are not sufficiently developed, moreover there is a lack of adequate research funding [31] which contribute to inaccessibility to medications.

Transport costs were the second most expensive direct cost of treatment for illness in both public and private hospitals. Villagers from rural areas were especially hard hit by high fuel prices and high associated transport costs, with this situation potentially limiting access to hospital health care facilities which are mostly located in towns and cities. This results in a significant welfare loss for rural and poor villagers seeking health care services. There is a role for government to play to ensure incentives are made available for doctors to relocate to primary health care centers based in rural areas. Otherwise, villagers will be adversely affected by high transport costs which results in inaccessibility to health services.

Income reductions caused by illness were very large. The majority of costs were indirect costs or loss of income from illness which was 97 % of the total cost for public patients and 95 % for private patients. These income losses were catastrophic with the economic burden varying little between illness morbidity and treatment modality. It has been recently observed elsewhere in a cost of cholera study in Bangladesh that indirect costs were over 75 % of total costs of illness [32]. These significant indirect costs of illness are routinely ignored by the health system and government.

Hidden and informal payments in public hospitals are widespread due to the long waiting times and poor management. Efficient functioning of any health system especially public hospitals which are frequently the only supply option for health care for the poor should not be dependent on bribery.

The public hospital quality of care was considered inferior compared to private hospitals due to the lack of an efficient and effective operating environment in public hospitals. This was manifested through informal payments, long waiting times and staff indifference and negligence. Policy makers should initiate behavioural training into the professional development programme for all of public health employees.

Other problems include the limited government health care budget, hospital management power and lack of information for consumers. Government has in recent times initiated some health care information services through mobile phones [33] but access to information is still uncertain due to the relatively high cost of mobile phones for the poorest. Budget limitations, hospital mis-management and a lack of human resources combine to further disadvantage poorer patients.

In the context of trying to achieve UHC whereby people do not suffer unreasonable financial hardship to pay for access to good quality health care services then a functioning and efficient insurance market for health care should be a major policy goal. Sadly this is far from the reality in Bangladesh. In this study only 10 patients (3.98 %) out of 252 patients had health insurance. Direct ‘out of pocket’ household expenditure accounts for an estimated 60 % of total spending on health care [34], with the remaining 40 % covered by public health care services [35]. These numbers strengthen the argument for health insurance.

Community based health insurance schemes have been initiated on a pilot basis in the past few years by non-government organizations. These have been fragmented, local and not successful mainly due to relatively high costs and low incomes. An investigation of micro health insurance systems within a public-private partnership should be undertaken. In 2007 the Ministry of Health and Family Welfare initiated a maternal health voucher to reduce the financial barriers to access to health care in pregnancy. The scheme did not attract any new providers into the market though increased satisfaction of public patients was expressed as a result of the higher level of services that the voucher system induced [36]. Given the extremely low incomes and relatively large out of pocket payments for health care there are strong equity arguments for the development of a central government health care financing model which incorporates health insurance.

There is strong evidence that health insurance provides financial protection by reducing ‘out of pocket’ spending. This study recommends health policy makers examine the establishment of a national health care insurance scheme which will provide protection from the catastrophic financial impacts of illness. Further, it has been shown elsewhere that universal health insurance supplemented by private insurance is successful in offsetting large informal payments [23].

### Study limitations

This study has several limitations - small sample sizes, non-representative sample (covering only one metropolitan area) and selection bias of patients between public and private hospitals.

The small sample size makes it difficult to find significant statistical relationships using advanced statistical methods, given these require larger sample sizes to ensure a representative sample of the population. The study is a snapshot of the city of Sylhet which may or may not be representative of health care delivery in other cities and towns in Bangladesh. Patient selection of either public or private hospitals could potentially bias the observed results, however several statistical tests were conducted to examine the extent of potential bias.

The poor in Bangladesh borrow money or sell household assets as their primary coping strategy to pay for the costs of treatment for illness [37]. This study did not consider the impact of high interest payments on borrowing money to pay for the cost of treatment for poor people. The implication is that the total cost of treatment is underestimated. In a few cases adult patients were accompanying by other adult family members, but the costs of these persons were not included in the cost calculations which again might underestimate the total cost of illness episodes.

## Conclusion

This study compared the total costs of treatment for illness between public and private hospitals in Bangladesh. It utilized different cost components (direct and indirect) and found that the total costs of outpatient treatment for illness were higher in the public sector compared to the private sector. Illness causes high indirect costs, and it was found that indirect costs comprised more than 90 % of total overall costs in both the public and private hospitals. This issue of very high indirect costs is important in a relatively poor country like Bangladesh. In the public sector, pro-poor policies such as ‘free medication’, and ‘low registration fees’ are very ineffective in reality to protect households from the financial burdens of illness. These policies cannot protect households from the large indirect costs of illness such as wage losses from long waiting times, the issue of informal payments to achieve better and/or quicker treatment and the low quality of health care services provided. Further policy actions to address these issues is urgently needed to stop and reverse the devastating financial effect of ill health and its treatment on the majority of Bangladesh citizens. Future research effort is needed to focus on equity issues associated with illness. A comprehensive national health insurance scheme should be investigated as a matter of urgency.

## Additional file

Additional file 1: Questionnaire. (PDF 209 kb)
