# Supplementary material for: Cost of illness for outpatients attending public and private hospitals in Bangladesh
Source: Int J Equity Health. 2016 Oct 10;15:167. doi: 10.1186/s12939-016-0458-x (PMC5057498; doi:10.1186/s12939-016-0458-x)
Supplement: Additional file 1: — Questionnaire. (PDF 209 kb) [file 12939_2016_458_MOESM1_ESM.pdf]

Health Related Contingent Valuation Study in Bangladesh

Department of Economics
Shahjalal University of Science & Technology, Sylhet-3114, Bangladesh.

Hospital Code:
MAG Osmani Medical College Hospital=1, Jalalabad Ragib-Rabeya Medical College & Hospital=2, Women's Medical College & Hospital=3, Northeast Medical College & Hospital=4, Others=5……………………………
Interviewer's Name:
Date:

I. Diagnosis of the current situation of Public Health Care services & elicitation of WTP values

1.1 Type of the disease for that visited to the PHC hospital?

____

1.2 By whom have you been examined?

1. Generalist ☐
2. Specialist ☐ Specify:______________________
3. Others ☐ Specify:______________________

2.1 Is this the first time that you come to here?

1 Yes ☐
2 No ☐ During the last 12 months, how many times did you visit the hospital?______

We have selected eight characteristics for the PHC services that you might be interested in, to be improved. We would like to know how do you evaluate, yourself, improvements on each of these characteristics. We are interested in the following eight characteristics:

**A. Geographical proximity of the PHC hospital from your home**	**B. Waiting time before seeing the doctor**
**C. Attitude of the PHC hospital's staff toward you**	**D. Being able to see the same health professional every time you come to the hospital**
**E. Being able to discuss your problem with the doctor and receive sufficient information about your health state and the prescribed treatment(s)**	**F. Being able to find the prescribed medicine(s) in the hospital**
**G. Being able to receive diagnostic test in the hospital**	**H. Your chance of recovery after visiting the hospital**

3. We are planning to improve each of these characteristics and we would like to know the importance of such improvements, <u>for you</u>, based on your needs and your preferences. Please, rank them <u>from the most important to be improved, for you, to the least important</u>.

	Rank	
1.		(The most important *for you*, to be improved)
2.		
3.		
4.		
5.		
6.		
7.		
8.		(The least important *for you*, to be improved)

4. Would you be willing to pay any amount of money in order to receive a higher quality service?

1. Yes ☐
2. No ☐

5.1 How did you come to here?

1. On foot.		4. By private car.	
2. By rickshaw.		5. By bus.	
3. By CNG		6. Others. Specify:	

***5.2 Total cost for that –**

☐

5.3 How long did it take you to reach here from your home (in minutes, approximately)?

☐

5.4 Do you consider that the hospital is situated ……

1. Very far from your home.		4. Close to your home.	
2. Far from your home.		5. Very close to your home.	
3. At an average distance from your home.			

5.5 For the existing "distance", what you consider about your payment to the PHC is:

1. Very Cheap		How much is it? Specify:
2. Cheap		How much is it? Specify:
3. Average		
4. Expensive		How much is it? Specify:
5.Very Expensive		How much is it? Specify:

5.6 To consider that the PHC hospital is "Very Close", it should be situated at which distance from your home (measured by travel time to the hospital)?

☐

5.7 Would you be willing to pay any amount of money more than what you already pay, in order to benefit from a hospital similar to this one and located "Very Close" to your home?

1. Yes ☐
2. No ☐ Why? ____(go to Q 6.1)

5.8 What is the maximum amount of money that you would be willing to pay, extra to what you currently pay, in order to have a hospital "Very Close" to your home; knowing that this extra amount of money will be paid at every visit?

WTP: ☐ How much can you afford? ☐

****6.1 How long did you wait before seeing the doctor (in minutes, approximately)?**

☐

6.2 Do you consider this "Waiting Time" as…..

1. Very long.		4. Not long.	
2. Long.		5. Not long at all.	
3. Average.			

6.3 For the existing "Waiting Time", what you consider about your payment to the PHC is:

1. Very Cheap		How much is it? Specify:
2. Cheap		How much is it? Specify:
3. Average		
4. Expensive		How much is it? Specify:
5.Very Expensive		How much is it? Specify:

6.4 What is the "Waiting Time" that you consider as "Not long at all" (in minutes, approximately)?

☐

6.5 Would you be willing to pay any amount of money more than what you already pay, in order to benefit from a "Waiting Time" which would be "Not long at all"?

1. Yes ☐
2. No ☐ Why? ____(go to Q 7.1)

6.6 What is the maximum amount of money that you would be willing to pay, extra to what you currently pay, in order to have a hospital with a "Waiting Time" that you estimate as "Not long al all"; knowing that this extra amount of money will be paid at every visit?

WTP: ☐ How much can you afford? ☐

****7.1 How do you describe the attitude of the hospital's staff toward you?**

1. Excellent.		3. Bad.	
2. Good.		4. Very bad.	

7.2 For the existing "attitude of the staff", what you consider about your payment to the PHC is:

1. Very Cheap		How much is it? Specify:
2. Cheap		How much is it? Specify:
3. Average		
4. Expensive		How much is it? Specify:
5.Very Expensive		How much is it? Specify:

7.3 Would you be willing to pay any amount of money more than what you already pay, in order to benefit from an "Excellent" attitude from the hospital Staff?

1. Yes ☐
2. No ☐ Why? ____(go to Q 8.1)

7.4 What is the maximum amount of money that you would be willing to pay, extra to what you currently pay, in order to benefit from an "Excellent" attitude from the hospital Staff; knowing that this extra amount of money will be paid at every visit?

WTP: ☐ How much can you afford? ☐

****8.1 Do you see the same health professional every time you come to the hospital?**

1. Always.		4. Never.	
2. Often.		5. This is my first visit.	
3. Rarely.			

8.2 For the existing "see the same professional", what you consider about your payment to the PHC is:

1. Very Cheap		How much is it? Specify:
2. Cheap		How much is it? Specify:
3. Average		
4. Expensive		How much is it? Specify:
5.Very Expensive		How much is it? Specify:

8.3 Would you be willing to pay any amount of money more than what you already pay, in order to be able to see the same health professional every time you come to the hospital?

1. Yes ☐
2. No ☐ Why? ____(go to Q 9.1)

8.4 What is the maximum amount of money that you would be willing to pay, extra to what you currently pay, in order to be able to see the same health professional every time you come to the hospital; knowing that this extra amount of money will be paid at every visit?

WTP: [ ] How much can you afford? [ ]

****9.1 How long did you stay with the doctor (in minutes, approximately)?**

[ ]

Please indicate your degree of agreement with each of the following statements. Circle one answer only for each statement.

	**Strongly Disagree**	**Disagree**	**Undecided**	**Agree**	**Strongly Agree**
**9.2 I stayed sufficient time with the doctor.**	1	2	3	4	5
**9.3 The doctor explained to me my health problem.**	1	2	3	4	5
**9.4 The doctor explained to me how to use the prescribed treatments.**	1	2	3	4	5
**9.5 The doctor explained to me what I should do to prevent (or not to complicate) my health state in the future.**	1	2	3	4	5
**9.6 The information that I get from the doctor was clear and sufficient.**	1	2	3	4	5

9.7 For the existing "Information from the Doctor", what you consider about your payment to the PHC is:

1. Very Cheap		How much is it? Specify:
2. Cheap		How much is it? Specify:
3. Average		
4. Expensive		How much is it? Specify:
5.Very Expensive		How much is it? Specify:

9.8 Would you be willing to pay any amount of money more than what you already pay, in order to be able to stay sufficient time with the doctor to discuss with him your health problem, receive sufficient and clear information about your disease and the prescribed treatment(s)?

1. Yes [ ]
2. No [ ] Why? ______________________________________(go to Q 10.1)

9.9 What is the maximum amount of money that you would be willing to pay, extra to what you currently pay, in order to be able to stay sufficient time with the doctor to discuss with him your health problem, receive sufficient and clear information about your disease and the prescribed treatment(s); knowing that this extra amount of money will be paid at every visit?

WTP: ☐ How much can you afford? ☐

****10.1 Did the doctor prescribe to you a medicament(s)?**

1. Yes ☐
2. No ☐ Go to Q 11.1

10.2 Within the range of money that you paid for registration in the PHC was (were) the medicament(s) available in the hospital?

1. Yes ☐ 2. No ☐ 3. Some of them ☐ 4. I don't know ☐

10.3 For the existing "Available Medicament(s)", what you consider about your payment to the PHC is:

1. Very Cheap		How much is it? Specify:
2. Cheap		How much is it? Specify:
3. Average		
4. Expensive		How much is it? Specify:
5.Very Expensive		How much is it? Specify:

10.4 Were you able to buy the prescribed medicine(s)?

1. Yes, all. ☐
2. Yes, ________ % ☐ Why? ____________________
3. No ☐ Why? ____________________

10.5 How do you buy the medicine(s)?

1. As doctor prescribed ☐
2. Pharmacy's preferences ☐
3. Your own preferences ☐
4. Some as doctor prescribed and some as pharmacy's preferences ☐
5. Some as doctor prescribed and some as own preferences ☐
6. Some as pharmacy's preferences and some as own preferences ☐

10.6 Do you prefer any brand for drug (medicine)?

1. Yes ☐ Which brand? Please Specify:
2. No ☐ Or , According to question number 10.5

10.7 Would you be willing to pay any amount of money more than what you already pay, in order to be able to find the prescribed medicine(s) "always" available in the hospital?

1. Yes ☐
2. No ☐ Why? ____________________(go to Q 11.1)

10.8 What is the maximum amount of money that you would be willing to pay, extra to what you currently pay, in order to be able to find the prescribed medicine(s) "always" available in the hospital; knowing that this extra amount of money will be paid at every visit?

WTP: [ ] How much can you afford? [ ]

****11.1 Did the doctor prescribe to you a diagnostic test(s)?**

1. Yes [ ]
2. No [ ] Go to Q 12

11.2 What type(s) of diagnostic test(s)?

1. Blood	[ ]	3. Ultra-sonogram	[ ]	5. X-Ray	[ ]
2. Urine	[ ]	4. ECG	[ ]	6. Others, Specify:	[ ]

11.3 Was (were) the diagnostic test(s) available in the hospital?

1. Yes [ ] 2. No [ ] 3. Some of them [ ] 4. I don't know [ ]

11.4 For the existing "Diagnostic Test", what you consider about your payment to the PHC is:

1. Very Cheap		How much is it? Specify:
2. Cheap		How much is it? Specify:
3. Average		
4. Expensive		How much is it? Specify:
5.Very Expensive		How much is it? Specify:

11.5 Were you able to test the prescribed diagnostic test(s)?

1. Yes, all. [ ]
2. Yes, some of it. [ ] Why? ____________________
3. No [ ] Why? ____________________

11.6 How much cost of diagnostic test(s) could you cover from your own income?

a. 0%	[ ]	d. 60%	[ ]
b. 20%	[ ]	e. 80%	[ ]
c. 40%	[ ]	f.100%	[ ]

11.7 Where you test the diagnostic test(s)?

1. In hospital [ ]
2. In diagnostic center [ ]

11.8 What you consider for choose the hospital/ diagnostic center for the diagnostic test(s)?

1. Doctor's preferences [ ]

2. Intermediary's preferences
3. Your own preferences
   Less expensive
   Hospital/ diagnostic center's good reputation
   Neat and Clean
   Accuracy
   Little Waiting Time

****12. Please indicate your degree of agreement with each of the following statements. Circle one answer only for each statement.**

	Strongly Disagree	Disagree	Undecided	Agree	Strongly Agree
**12.1 I'm usually recovered after being examined by the doctor of the hospital.**	1	2	3	4	5
**12.2 Many times I need to go to a private clinic to be re-examined by a better doctor because I wasn't recovered.**	1	2	3	4	5
**12.3 The doctor who examined me was a good doctor who knows what he is doing.**	1	2	3	4	5
**12.4 I believe that private doctors are more competent.**	1	2	3	4	5
**12.5 I would actually prefer to go to a private clinic.**	1	2	3	4	5

12.6 For the existing "chance of recovery", what you consider about your payment to the PHC is:

1. Very Cheap		How much is it? Specify:
2. Cheap		How much is it? Specify:
3. Average		
4. Expensive		How much is it? Specify:
5.Very Expensive		How much is it? Specify:

12.7 Would you be willing to pay any amount of money more than what you already pay, in order to be examined by more competent doctors and to have a higher chance of recovery?

1. Yes
2. No   Why? ________________________________(go to Q 13.1)

12.8 What is the maximum amount of money that you would be willing to pay, extra to what you currently pay, in order to be examined by more competent doctors and to have a higher chance of recovery; knowing that this extra amount of money will be paid at every visit?

WTP: How much can you afford?

13.1 Number of days without doing regular work -

***13.2 Income loss for those days-**

****14.1 Between the eight discussed characteristics, select the three characteristics that you estimate as the most important, <u>for you</u>, to be improved.** (Put X in the corresponding cases).

1. The geographical proximity of the hospital from your home----------------------------------
2. Waiting time before seeing the doctor--
3. Attitude of the hospital-staff toward you--
4. Being able to see the same health professional every time you come to the hospital--------
5. Being able to discuss your problem with the doctor and receive sufficient & clear information about your health state and the prescribed treatment(s)-----------------------------
6. Being able to find the prescribed medicine(s) in the hospital----------------------------------
7. Being able to test the diagnostic test(s) in the hospital ---
8.Your chance of recovery after visiting the hospital ---

14.2 What is the maximum amount of money that you would be willing to pay, extra to what you currently pay, in order to have these three characteristics improved simultaneously; knowing that this extra amount of money will be paid at every visit?

WTP: How much can you afford?

15.1 How much did you pay the consultation (only the consultation; i.e., without the medicines)?

15.2 Do you consider this as:

1. Very Cheap		How much is it? Specify:
2. Cheap		How much is it? Specify:
3. Average		
4. Expensive		How much is it? Specify:
5.Very Expensive		How much is it? Specify:

15.3 Why did you choose to come to this hospital?

____

15.4 Do you go to other PHC hospital(s) or private clinic(s) better than this one?

1. Yes
2. No Go to Q. 16.1

15.5 What aspect(s) is (are) better in the other hospital or private clinic?

____

15.6 How much do you pay the medical consultation in the other hospital or private clinic?

16.1 Now it became clearer for you what do we mean by improving the primary health care services. I would like to re-ask you a question that I asked you in the beginning. Would you be willing to pay anything in order to receive a better service?

1 Yes ☐ Go to question 17
2 No ☐ Why? ____

16.2 Could you please tell which one(s) of the listed reasons best explain why you are not willing to pay for an improvement in the quality of the offered services?
[READ and tick column 1then column 2 if several ANSWERS]

1. I can't afford it		
2. I already pay enough		
3. I prefer other ways of paying		
4. It's my right to get the best quality		
5. Government should allocate more resources to the health sector		
6. Only financially comfortable people should pay		
7. I'm not concerned		
8. Other (please specify:________________________________)		

II. Socioeconomic and Demographic Information:

17. Patient's Name:

18. Patient's relation with the head of the household:

1. Self		4. Mother		7. Niece/ Nephew	
2. Child		5. Spouse		8. Brother/ Sister	
3. Father		6. Grand Child		9. Other	

19. Sex:

Male ☐ Female ☐

20. In which year were you born? /__/__/__/__/ or age /__/__/

21. Where are you coming from (name of the city/village)? ______________________

22. What is your marital status?

1 Married		3 Divorced	
2 Widowed / widowered		4 Single	

23. How many persons are there in your household (those who live together in the same home or eat together)?

24. How many persons (children, parents, etc.) are dependent on your income?

25. Number of schooling years:

26. What is your main activity? (patient's activity or the one responsible of the patient (the one who paid for the patient & answered the questions; ex. mother):

27. What is your household monthly-income (this includes the revenues of all the persons in the household):

28. Do you have any type of health insurance?

1 Yes		Specify: ____________________________________
2 No		

<u>For the interviewer</u>

29. How long did the interview last? _____ minutes
